# Targeting the Endocannabinoid System Present in the Glioblastoma Tumour Microenvironment as a Potential Anti-Cancer Strategy

**DOI:** 10.3390/ijms25031371

**Published:** 2024-01-23

**Authors:** Mendhi Henna Dasram, Pavesan Naidoo, Roderick B. Walker, Sandile M. Khamanga

**Affiliations:** Division of Pharmaceutics, Faculty of Pharmacy, Rhodes University, Makhanda 6139, South Africar.b.walker@ru.ac.za (R.B.W.)

**Keywords:** endocannabinoid system, glioblastoma tumour, glioblastoma tumour microenvironment, targeted nanotherapeutics

## Abstract

The highly aggressive and invasive glioblastoma (GBM) tumour is the most malignant lesion among adult-type diffuse gliomas, representing the most common primary brain tumour in the neuro-oncology practice of adults. With a poor overall prognosis and strong resistance to treatment, this nervous system tumour requires new innovative treatment. GBM is a polymorphic tumour consisting of an array of stromal cells and various malignant cells contributing to tumour initiation, progression, and treatment response. Cannabinoids possess anti-cancer potencies against glioma cell lines and in animal models. To improve existing treatment, cannabinoids as functionalised ligands on nanocarriers were investigated as potential anti-cancer agents. The GBM tumour microenvironment is a multifaceted system consisting of resident or recruited immune cells, extracellular matrix components, tissue-resident cells, and soluble factors. The immune microenvironment accounts for a substantial volume of GBM tumours. The barriers to the treatment of glioblastoma with cannabinoids, such as crossing the blood–brain barrier and psychoactive and off-target side effects, can be alleviated with the use of nanocarrier drug delivery systems and functionalised ligands for improved specificity and targeting of pharmacological receptors and anti-cancer signalling pathways. This review has shown the presence of endocannabinoid receptors in the tumour microenvironment, which can be used as a potential unique target for specific drug delivery. Existing cannabinoid agents, studied previously, show anti-cancer potencies via signalling pathways associated with the hallmarks of cancer. The results of the review can be used to provide guidance in the design of future drug therapy for glioblastoma tumours.

## 1. Introduction

The latest version of the Central Nervous System (CNS) tumour classification published by the World Health Organization (WHO) summarises updates from the Consortium to inform molecular and practical approaches to CNS tumour taxonomy work [1]. The classification of tumours by the World Health Organization is an important tool for the diagnosis and treatment of brain tumours, including glioblastoma multiforme, the most common and aggressive malignant primary brain tumour in adults [2]. GBMs are highly invasive and diffuse tumours characterised by rapid proliferation, angiogenesis, and resistance to therapy [3]. Despite significant progress in our understanding of GBM biology and the development of novel therapeutic approaches, the prognosis for GBM patients remains poor [4]. 

One of the main challenges in treating GBM is the highly complex and dynamic nature of the GBM tumour microenvironment, which plays a crucial role in tumour growth, invasion, and resistance to therapy [5]. The GBM tumour microenvironment comprises various cell types, including tumour cells, astrocytes, microglia, endothelial cells, and immune cells, as well as extracellular matrix components, growth factors, and cytokines [6,7]. The interactions between these components create a highly heterogeneous and dynamic environment that facilitates tumour progression and adaptation to therapy [8]. Recent studies have highlighted the importance of the macroenvironment and microbiome in GBM pathogenesis and treatment [9,10]. Tumours release factors that drive the orchestration of an environment in the host that involves the crosstalk between multiple distal compartments at places beyond tumour beds [11]. Systemic alterations include changes in the bone marrow’s functioning, where myelopoiesis is especially heavily altered in the presence of a tumour [12,13]. Distal hormonal signals and inflammatory mediators generated through interactions with commensal microorganisms also facilitate the formation of premetastatic niches where disseminated tumour cells call home, lay dormant, and eventually develop into growing metastatic [14,15,16,17]. Together, these inflammatory, tumour-promoting pro-metastatic networks form a systemic “macroenvironment” in tumour-bearing hosts that influence both the function of distant tissues and the tumour itself [18]. In the current era of personalised medicine, the identification and comprehensive understanding of cancer’s pathophysiological mechanisms are crucial for tailoring therapies based on grade, histological features, molecular subtypes, aggressiveness, and treatment response.

The endocannabinoid system (ECS) is a widespread neuromodulatory network that plays a role in the developing nervous system and mature nervous system by modulating network function and neuronal activity [19]. G-protein coupled cannabinoid receptors including the canonical receptor subtypes cannabinoid receptor type 1 (CB1-R) and cannabinoid receptor type 2 (CB2-R), endogenous cannabinoids known as endocannabinoids (e.g., anandamide and 2-arachidonoylglycerol), and the proteins that synthesize and degrade endocannabinoids, fatty acid amide hydrolase (FAAH) and monoacylglycerol lipase (MAGL), comprise the endocannabinoid system [20,21]. In addition to the enzymes involved in the biosynthesis and degradation of endocannabinoids, the other “non-canonical” extended signalling network of the ECS include receptors GPR55 and PPARα, inotropic cannabinoid receptors (TRP channels), protein transporters (FABP family), and other fatty acid derivatives [22,23,24,25]. While cannabinoid receptors are present in most tissues, CB1-R is primarily and largely found in the CNS, moderately found in adipose, endocrine, lymphoid, and female tissues and in smaller amounts found in other tissues [26]. The endocannabinoid system has emerged as a potential target for treating GBM [27]. The ECS is a complex signalling system that plays a crucial role in maintaining homeostasis in the body [28]. The ECS involves various physiological processes, including pain modulation, appetite, mood, and immune function [29]. Recent evidence suggests that the ECS is dysregulated in GBM, and that its manipulation could represent a promising therapeutic strategy [30,31]. In particular, CB1-R and CB2-R are expressed in GBM cells and the tumour microenvironment, including immune cells and endothelial cells [32,33,34]. Activation of these receptors has been shown to induce antitumour effects in preclinical studies, including inhibition of tumour cell proliferation, migration, invasion, and angiogenesis [35].

Moreover, cannabinoid-induced apoptosis has been reported in GBM cells [36]. Despite the promising preclinical data, the clinical translation of most cannabinoids for the treatment of GBM faces significant challenges, including poor water solubility, limited bioavailability, and poor pharmacokinetics [37]. These limitations have led to the investigation of novel drug delivery systems, including nanocarriers, which have shown promising results in preclinical studies [38]. Nanocarriers are nanoscale drug delivery systems that can encapsulate hydrophobic drugs, such as cannabinoids, and protect them from degradation, enhance their solubility, and increase their bioavailability [39].

The complex and dynamic nature of the GBM microenvironment presents significant challenges developing effective therapies. This paper reviews recent progress in the dysregulation of the ECS in GBM and its potential as a therapeutic target, together with the development of novel drug delivery systems, including nanotechnology, to offer a promising approach to the future treatment of this fatal disease.

## 2. Classification of Glioblastoma Tumours

The glioblastoma multiforme tumour is one of the most aggressive cancers discovered in the human CNS and the most common primary brain tumour in adults [40]. It is defined as a central nervous system tumour that displays immunohistochemical and ultrastructural evidence of glial differentiation. GBM accounts for 45.2% of malignant primary brain and central nervous system tumours [41]. While glioma is a general term which describes primary brain tumours, these nervous system tumours are classified according to their presumed cell of origin [42]. The central nervous system includes the brain and spinal cord and the peripheral nervous system originating from neuroglia cells containing various types of glial cells, including astrocytes, oligodendrocytes, ependymal cells, and microglia cells [43]. Table 1 shows the nomenclature-derived cell of origin of associated brain tumours and the WHO grade classification of tumours. The WHO Classification of Tumours Editorial Board released a malignancy scale of gliomas graded from I to IV and defined by increasing aggressiveness and histological features [44]. Grade I tumours are benign and possess a relatively slow proliferation rate. Grade II tumours also have a slow growth rate, a significant degree of cellular differentiation, and diffusive growth into normal brain parenchyma, which makes them more prone to malignant progression. Grade III tumours have a characteristically large amount of atypia and mitotic cells combined with higher cellular density. Grade IV tumours possess the characteristics of grade III tumours, in addition to either or both microvascular proliferation and pseudo palisading necrosis. While grade IV tumours such as glioblastoma are the most malignant, they are also the most common primary brain tumour [45]. 

**Figure 1:**
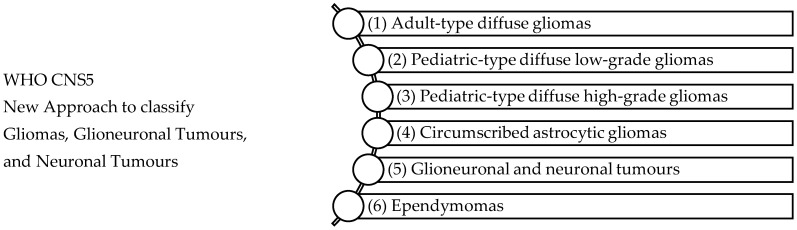
WHO CNS5 New Approach to classify Gliomas, Glioneuronal Tumours, and Neuronal Tumours [49]. The WHO CNS5 classification categorizes gliomas, glioneuronal tumours, and neuronal tumours into six distinct families: (1) Adult-type diffuse gliomas are the most common brain tumours constituting the bulk of adult neuro-oncology including glioblastoma (IDH-wildtype), astrocytoma (IDH-mutant), and oligodendroglioma (IDH-mutant and 1p/19q-codeleted), (2) Paediatric-type diffuse low-grade gliomas usually present favourable outcomes and include diffuse astrocytoma (MYB- or MYBL-1-altered), angiocentric glioma, polymorphous low-grade neuroepithelial tumour of the young, and diffuse low-grade glioma (MAPK pathway-altered), (3) Paediatric-type high-grade gliomas are typically aggressive tumours and include diffuse midline glioma (H3 K27-altered), diffuse hemispheric glioma (H3 G34-mutant), diffuse paediatric-type high-grade glioma (H3-wildtype and IDH-wildtype), and infant-type hemispheric glioma, (4) Circumscribed astrocytic gliomas are termed “circumscribed” considering their more contained growth pattern when compared to “diffuse” tumours, (5) Glioneuronal and neuronal tumours, and (6) Ependymal tumours.

The WHO glioma classifications have been influential in associating genomic or molecular variations to clinical phenotypes of gliomas [47]. Previously, primary CNS tumours were classified according to histological parameters and assigned a grade from I to IV. Figure 1 shows the fifth edition of the WHO Classification of Tumours; the classification is revised to combine signature molecular genetic alterations with classic histology resulting in an integrated diagnosis where the histopathological name of the tumour is followed by the genetic features [48,49]. Molecular genotypic key markers are explained in Table 2 and include iso-citrate dehydrogenase (IDH), p53 mutations, ATP-dependent helicase (ATRX), 1p/19q chromosomal deletion, and Lys-27-Met mutations in histone 3 (H3K27M). The WHO defines glioblastoma (grade IV), IDH-wildtype as a diffuse, astrocytic glioma that is IDH-wildtype and H3-wildtype with one or more characteristic histological or genetic featuring including necrosis, EGFR gene amplification, microvascular proliferation, TERT promoter mutation, or +7/−10 chromosome copy-number changes [49]. Gliomas, glioneuronal and neuronal tumours are the most varied and common tumours disturbing the parenchyma of the CNS [49].

Since the addition of cellular features, molecular features, and grade of malignancy in the classification of gliomas, formerly “glioblastoma multiforme” is currently classified glioblastoma, IDH-wildtype to distinguish from grade 4 astrocytoma, IDH-mutant [3,56,57]. The cIMPACT-NOW initiative, which stands for Consortium to Inform Molecular and Practical Approaches to CNS Tumour Taxonomy, is a collaborative effort to evaluate and recommend changes to future classifications of central nervous system tumours [58]. cIMPACT-NOW aims to enable the sharing and agreement on new diagnostic information that can be useful in identifying CNS tumours and to determine how this information can be practically used in the classification of such tumours in the future [59]. While the WHO classification update process is the primary mechanism for international brain tumour classification, cIMPACT-NOW is anticipated to impact selected tumour types and during periods between the WHO classification updates. The initiative is not intended to supplant the existing WHO classification, nor is it officially part of the WHO process. Instead, the cIMPACT-NOW updates are intended to provide possible guidelines for practising diagnosticians and guideposts for future WHO classification updates.

Despite many attempts to successfully treat GBM, it remains an incurable disease with a poor prognosis and median survival time of approximately 15 months [60]. The short survival rate of GBM is attributed to the expansion of the primary brain tumour through diffuse invasion which results in disruption of the healthy brain architecture [61,62]. The classification of glioblastoma is shown in Table 3.

**Table 3:** The landscape of GBM.

Landscape of Glioblastoma Tumours		
Etiology	While the etiology of most glioblastomas remains unknown, a small population is inherited as part of genetic tumour syndromes. Although environmental factors such as non-ionising radiation from mobile phones and occupational exposures have been investigated as potential causes, the results remain negative or inconclusive.	
Epidemiology	GBM occurs mostly in older adults, with peak incidence in patients between the ages 55–85 years old. The male to female incidence ratio of GBM is 1.4, which shows a higher occurrence amongst males compared to females. In the United States the M:F ratio is 1.60:1 and 1.28:1 in Switzerland. A substantial difference in incidence rates of GBM by race and ethnicity have been demonstrated by previous studies. Consistent finding shows that incidence of GBM is the highest amongst the Caucasian population compared to African or Asian populations. While approximately 80% of all malignant brain tumours are gliomas, an estimated 70% of the reported gliomas are GBM. The annual incidence of newly diagnosed cases has been reported to be between 3 and 5 per 100,000 inhabitants.	[49,63,64]
sLocalisation	GBM is often centred in the subcortical white matter and deeper grey matter of the cerebral hemispheres which affects all cerebral lobes. When tumour infiltration occurs, it extends into the adjacent cortex and into the contralateral hemisphere through the corpus callosum. GBM has also been found to affect the brainstem, cerebellum, and spinal cord. The sites of glioma tumours are shown in Figure 2.	
Histopathology	Cytological atypia, high cellularity, and mitotic activity coupled with necrosis are the key features required for histological diagnosis of GBM.	[65]
Molecular classification	High cellular proliferation and angiogenesis resulting in rapid tumour growth and necrosis. High migration and invasive properties. Glioma stem-like cells partially account for high resistance to therapy and recurrence rates.	[57]
Symptoms	Pain, difficulty communicating, perceived cognition, seizures, weakness, fatigue, and aphasia.	[66]
Genomic profiling	The importance of O6-methylguanine-DNA methyltransferase (MGMT), GATA binding protein 6 (GATA6), and caspase-8 (CASP8) gene methylation was demonstrated in a study where high methylation frequency showed a correlation between heterogeneity of GBM epigenome and patient outcome. The promoter region’s methylation is the mechanism that affects gene expression in tumours, and newly identified epigenetically modified genes play a role in glioblastoma genesis and understanding patient outcome and the differences between long term and short-term survivors. Elucidation of the molecular differences of GBM in long-term survivors in a study investigated the genome and transcriptome-wide molecular profiling of GBM samples from 94 patients. The study consisted of 28 long-term survivors (>36 months survival), 20 short-term survivors (<12 months survival) and 46 intermediate survival patients. The gene expression profile of long-term survivors was linked to IDH1/2 mutation and associated with more MGMT-methylated tumours.	[67]

**Figure 2:**
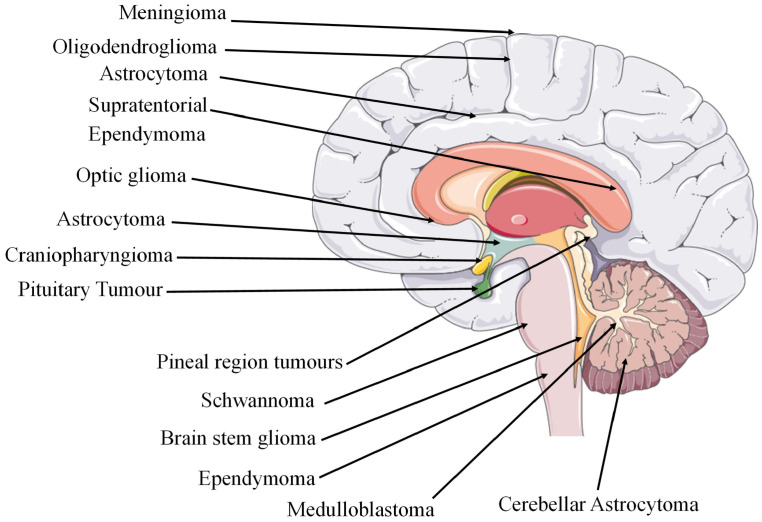
Sites of cancerous tumours in the brain region [68]. The figure contains modified images from Servier Medical Art (https://smart.servier.com, accessed on 5 July 2023) licensed by a Creative Commons Attribution 3.0 Unported License.

## 3. GBM Tumour Microenvironment, Macroenvironment and Microbiome

**Figure 3:**
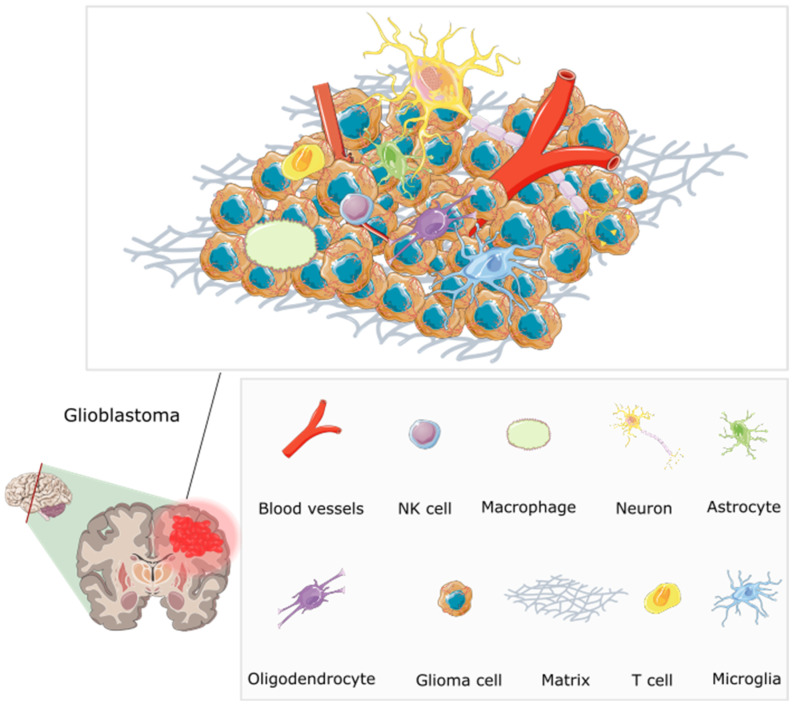
The multifaceted microenvironment of a glioblastoma, encompassing an intricate web of abnormal blood vessels, tumour cells, extracellular matrix, and immune modulators [71]. The blood vessels are twisted and irregular, characterised by hyperpermeability due to overexpression of pro-angiogenic factors including VEGF (vascular endothelial growth factor). They lack typical blood–brain barrier functions, and molecules can be seen leaking through. Tumour cells are densely packed, and interspersed immune cells such as T cells and macrophages are visible. Immune modulators, including cytokines and chemokines, are present, influencing the behaviour of tumour and immune cells. The extracellular matrix shows abnormal characteristics contributing to tumour growth. Redrawn with permission from Simon, T., Breaking through the Glioblastoma Micro-Environment via Extracellular Vesicles; published by Oncogene, 2020. CC-BY 4.0. http://creativecommons.org/licenses/by/4.0/ (accessed on 5 June 2023). The figure contains modified Images from Servier Medical Art (https://smart.servier.com, accessed on 5 July 2023) licensed by a Creative Commons Attribution 3.0 Unported License.

Although extensive research has been conducted on the genetics of GBM, the tumour microenvironment (TME) has often been overlooked and underestimated in the development of therapeutic interventions. The TME controls tumour growth and invasion in GBM and is composed of components of both the tumour niche and the organismal milieu [69]. The TME is made up of various cell types and soluble factors that influence the tumour growth, immune evasion, angiogenesis, invasion, and drug resistance including bone marrow-derived macrophages, myeloid cells (e.g., resident microglia), tissue-resident cells (e.g., astrocytes and neurons), fibroblasts, pericytes, and endothelial cells which are surrounded distinctly by an extracellular matrix (ECM). Cell-to-cell signalling plays a crucial role in the growth of tumours due to the various genetic mutations and changes to chromosomes that disrupt the normal flow of cell signalling via growth factors or cytokines. This signalling occurs between near and distant cells in the TME or between cells that are hypoxic and normoxic within a tumour. The ‘go or grow’ behaviour of glioma cells, deciding whether to migrate or proliferate, may be influenced by several factors in their surrounding environment. In a study where the TME was mathematically modelled using a reaction-diffusion equation to understand and simulate how various components involved in tumour growth interact and spread, the authors found that the model presented a good agreement with the experimental laboratory data. The model was validated by comparing a transwell assay of microglia secreting transforming growth factor beta to stimulate glioma cells in a laboratory and the mathematical model. The authors hope that developing these models will allow for the exploration of various hypothetical scenarios, and the prediction of tumour behaviour under different conditions to supplement the sparse experimental data, highlighting the significance of the tumour microenvironment. [70]. Figure 3 shows the inefficient microcirculation and complex interplay between the various TME components, highlighting challenges in treating glioblastomas.

The tumour macroenvironment of GBM refers to the systemic factors that affect the tumour development and response to therapy, such as the blood–brain barrier permeability, hormonal status, metabolic state, and microbiota. Tumours release factors that lead to the formation of an environment in the host that involves communication between various distant compartments beyond the immediate tumour area. This environment can cause systemic changes, such as alterations in bone marrow function, particularly in myelopoiesis, in the presence of tumours. Unlike emergency myelopoiesis which is induced by acute infections, in cancer normal myeloid cell differentiation is also redirected from its intrinsic pathway of differentiation of terminal differentiation to mature myeloid cells including dendritic cells, macrophages, and granulocytes towards a pathway caused by signals derived from the TME that generates pathologically activated immature and immunosuppressive cells [18]. This leads to the accumulation of myeloid precursors, which impairs dendritic cell-mediated antigen presentation and macrophage-dependent cytotoxic protective activity. Initially, mobilised immature myeloid cells may not have immunosuppressive properties, but they could contribute to tumour-promoting inflammation and neovascularisation. However, as the tumour progresses, the constantly produced immature myeloid cells are exposed to multiple tumour-derived factors that transform them into potent suppressors of protective immune responses. These heterogeneous immature myeloid cells that suppress antitumour T-cell responses through various mechanisms are generally referred to as myeloid-derived suppressor cells (MDSCs) and the accumulation of regulatory T cells and MDSCs are among the mechanisms employed by GBM to induce immunosuppression [72,73]. 

Hormonal signals and inflammatory mediators generated by interactions with commensal microorganisms, which not only maintain homeostasis at mucosal surfaces and provide a first line of defence against invading pathogens, but also play a role in the development and function of the immune system, and can also promote the development of premetastatic niches where disseminated tumour cells can establish and eventually grow into metastatic tumours when the balance in the composition of the microbiota is altered in cancer [74]. The microbiota, which varies among individuals, can affect the production of cytokines including TNFα, IL-6, and IL-17, which in turn affect the immune environment and tumour growth [75]. Therefore, it is important to consider genetic variation and the composition of the commensal microbiota when developing personalised cancer treatments [76]. These prometastatic networks form a systemic “macroenvironment” in tumour-bearing hosts that can affect the function of distant tissues and the tumour itself [77]. The tumour microbiome is the collection of microorganisms that reside in and around the tumour site, which may modulate inflammation, tumour immunity, and drug metabolism. Although previously thought to be a sterile organ, the detection of microbial sequences in pathological and non-pathological human brain samples were observed and revealed that *Proteobacteria* was the most abundant phylum present in all brain samples while members of the *Firmicutes*, *Proteobacteria*, and *Bacteroidetes* were identified in human and mouse brains under non-infectious conditions [78,79]. In a study which validated bacteria in a large group of 1010 tumour samples and 516 normal adjacent tissues, the tumour microenvironment was analysed in seven solid tumour types which included bone, melanoma, ovary, breast, pancreas, lung cancer and glioblastoma multiforme, and revealed bacterial lipopolysaccharide (LPS) and 16S *rRNA* present in all tumour types [80]. Currently, ongoing clinical trials investigating the role of tumour microbiome in different cancer types are being conducted [81]. A study examining the microbiome of 40 glioblastoma samples found that they had different bacterial profiles when compared to normal brain tissue. Bacterial DNA was detected in over 40% of the samples and a total of 22 bacterial taxa were identified on GBM tumours by 16S *rRNA* sequencing. Bacteria has been shown to affect cancer development and progression in various ways, such as cellular transformation caused by mutations or damage to host DNA, influencing biological processes related to tumour growth, invasion and metastasis through metabolism or interactions with immune cells, and interfering with the function of the tumour suppressor protein p53 [82,83]. Researchers hypothesised that the gut microbiome may influence the response of GBM patients to anti-cancer immunotherapy [84,85]. Using a unique model in which mice were colonised with human microbial communities from five different healthy donors, the authors of a recent study found that the human microbial communities in the GI tract of the mice influenced the response to immunotherapy, with some exhibiting a beneficial response and others being nonresponsive [86]. This is the first study to examine how human microbial communities influence the growth and response to therapies in a preclinical model of GBM. The findings suggest that personalised approaches to GBM treatment that consider the gut microbiome may be necessary for optimal response to immunotherapy [87]. 

Unravelling glioblastoma’s complexity demands a shift from local microenvironment to a systemic perspective, where the host macroenvironment becomes a crucial conductor of tumour progression [88]. The TME and tumour evolution are intertwined with a complex interplay of local and systemic factors. Local factors, including the immune response, ECM, and adaptive angiogenesis, exert a profound influence on TME composition and subsequent tumour progression [89]. Additionally, a plethora of systemic host factors, such as intestinal dysbiosis, stress-associated neurotransmitters/neurohormones, metabolic aberrations in both host and tumour, latent infections, and surgical/physicochemical stimuli, can significantly impact treatment response [90]. These factors can further activate the hypothalamic-pituitary-adrenal axis, potentially exacerbating metastatic risk. This intricate interplay highlights the need for a comprehensive and multifaceted approach to therapeutic intervention, considering the interplay between the TME and systemic factors.

## 4. Hallmarks of GBM

**Figure 4:**
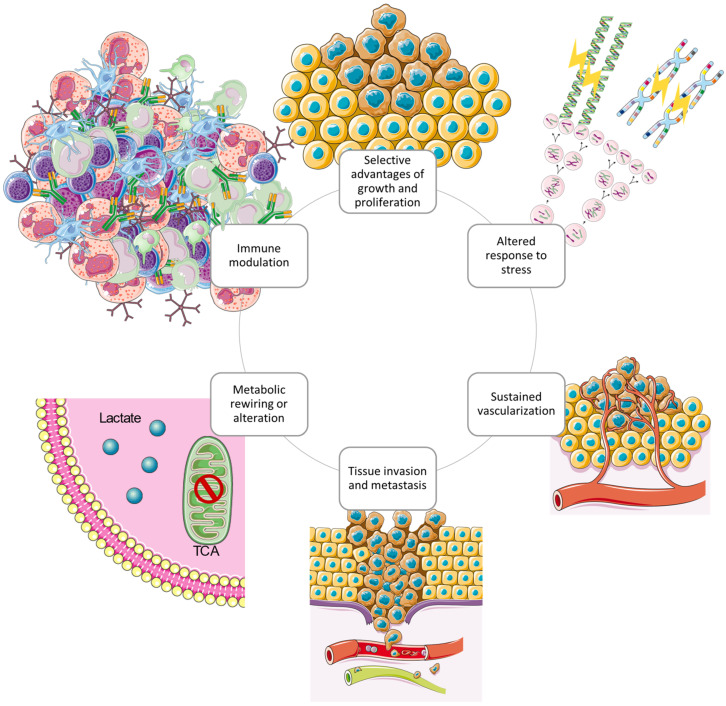
The circular structure of the interconnected cancer hallmarks shows the promotion and maintenance of the GBM tumour microenvironment. The hallmark capabilities exerted by GBM cells cooperate to create a tumour microenvironment which supports survival and progression using several strategies [95]. Redrawn with permission from Torrisi, F., The Hallmarks of Glioblastoma: Heterogeneity, Intercellular Crosstalk and Molecular Signature of Invasive-ness and Progression; published by Biomedicines, 2022. Creative Commons CC BY http://creativecommons.org/licenses/by/4.0/ (accessed on 25 June 2023). The figure contains modified Images from Servier Medical Art (https://smart.servier.com, accessed on 5 July 2023) licensed by a Creative Commons Attribution 3.0 Unported License.

In the last half century of advancements in cancer research, a rich and multifaceted body of knowledge has emerged and continues to emerge that has revealed cancer to be a dynamic disease involving changes in the human genome and indicates that tumourigenesis is a multistep process reflecting genetic alterations that guide the transformation of normal human cells into highly malignant derivatives [91,92]. In the year 2000, Hanahan et al. proposed that the extensive catalogue of cancer cell genotypes was an expression of six essential alterations in cell physiology that collectively control malignant growth, which they called the hallmarks of cancer [93]. The six hallmarks of cancer suggested were evading apoptosis, insensitivity to anti-growth signals, self-sufficiency in growth signals, sustained angiogenesis, tissue invasion and metastasis, and a limitless replicative potential. The six acquired capabilities of cancer are physiological changes occurring during tumour development which breach anti-cancer defence mechanisms present in tissues and cells and can be found in common in all types of human tumours. As the knowledge of cancer mechanisms progressed, Hanahan et al. have expanded the six distinct hallmark capabilities to eight since other facets of the disease have emerged as potential refinements [94]. Therefore, in addition to the existing hallmark capabilities, phenotypic plasticity and disrupted differentiation emerged as discrete hallmark capabilities. Senescent cells have also shown to be functionally important cell types in the tumour microenvironment. Also, nonmutational epigenetic reprogramming and polymorphic microbiomes both play a role as enabling characteristics which facilitate the acquisition of hallmark capabilities [67]. An excellent review on the hallmarks of glioblastoma shows how the newly proposed hallmarks fit well to describe GBM since the overlap between biological processes is more evident in GBM compared to other cancer diseases [95]. According to the glioma stem-cell-hypothesis model, glioma initiating cells (GBM stem cells) are characterised by the distinct hallmark capabilities that are involved in GBM pathogenesis and progression. Specifically, the GBM hallmark capabilities presented in Figure 4. 

Due to the highly mutated genome of GBM, several interconnected pathways involving epigenetic factor contribution, inactivation of tumour-suppressor genes and the activation of oncogenes creates an imbalance in intracellular signals which results in dysregulation of key signalling pathways involving cell growth, apoptosis, survival, and proliferation [96,97]. During uncontrollable proliferation cancer cells readjust energy metabolic processes, shifting the anabolic and catabolic balance and driving bioenergy and biological molecules essential for cell growth, self-renewal, and metabolic homeostasis to facilitate proliferation and maintenance of a malignant phenotype [98]. Cannabinoids have demonstrated metabolic reprogramming capabilities in cancer through signalling pathways and enzyme regulation, as well as mitochondrial function interference which hinders metabolism to mediate apoptosis and tumour autophagy [99,100].

## 5. Cannabinoids as a Promising Adjuvant in the Treatment of GBM

The endocannabinoid system which includes endocannabinoids and the enzymes that synthesise and degrade them, and the transporters and G-protein coupled receptors involved in their signalling have been found in glioblastoma cells [31,101]. The ECS is a homeostatic system that uses lipid-derived signalling molecules to regulate a wide range of physiological functions [102]. Studies have shown high levels of cannabinoid receptors, CB1-R and CB2-R, as well as the transient receptor potential vanilloid 1 receptor expressed on glioblastoma cells which are regulated by genetic and epigenetic mechanisms [103]. Although the expression levels obtained by immunohistochemistry are heterogeneous and dependent on the age of the patient and the histopathological origin of the brain tumour cells, CB2-R expression has been positively correlated with tumour grade and upregulated in most glioblastomas [104]. According to immunohistochemical analysis, both CB1 and CB2 receptors were detected in around 38% and 54% of glioblastoma endothelial cells, respectively [105]. CB2-R expression levels were found to be higher than CB1 in glioblastoma tissues. These findings suggest that selective CB2-R agonists could potentially serve as crucial targets for the treatment of glioma. The term “cannabinoids” originally described bioactive constituents of the *Cannabis sativa* plant. It is now an umbrella term covering a broad range of compounds sub sectioned into the synthetic cannabinoids, the phytocannabinoids, and the endogenous cannabinoids, most of which are ligands which bind to endogenous cannabinoid (e.g., CB1-R and CB2-R) and other G-protein coupled receptors [105,106]. The endogenous cannabinoids are naturally occurring lipid mediators that are synthesised from the membrane phospholipids of cells [107].

Table 4 provides an overview of the main classes of cannabinoids: classical cannabinoids, non-classical cannabinoids, aminoalkylindoles, and eicosanoids [108]. For each class, the table summarises the structural characteristics, formulation strategies, and metabolism. This information can be used to understand the unique properties of each class of cannabinoids.

Cannabinoid receptor activation can lead to the modulation of downstream signalling pathways in glioblastoma cells, including the PI3K/Akt/mTOR pathway, the MAPK/ERK pathway, and the c-Jun N-terminal kinase (JNK) pathway [123,124]. The activation of these pathways can have diverse effects on cell proliferation, differentiation, survival, and migration, depending on the specific context and the balance of signalling inputs. In addition to the modulation of signalling pathways, cannabinoids can also regulate gene expression in glioblastoma cells. For example, some cannabinoids such as THC, have the capacity to influence the expression of the tumour suppressor gene for p53 [125], while inhibiting the expression of genes involved in cell cycle progression and angiogenesis, such as cyclin A and D1 and VEGF [126,127,128,129]. The molecular mechanisms of cannabinoid action in glioblastoma are complex and involve both receptor-dependent and -independent pathways. In addition to the modulation of the ECS and downstream signalling pathways, cannabinoids can also interact with other targets, such as ion channels, other G protein-coupled receptors, and nuclear receptors [130,131,132,133]. An increasing number of preclinical models and clinical studies have investigated the anti-cancer effects of cannabinoids on a variety of cancers [134]. Reports have shown a dysregulation of cannabinoid receptors and endogenous ligands present in the tumour microenvironment of cancerous tumours; however, the ‘endocannabinoid’s system role suggests both pro-tumourigenic and anti-cancer effects based on the type and site of cancer [135]. Some authors attribute these inconsistencies to an incomplete elucidation of this complex biological system, the bystander effect or heterogeneity of receptors present in the disease state [103]. An important systematic review that the 2017 National Academy of Sciences committee used to review the health effects of cannabis focused on gliomas, and identified 2260 studies, of which 35 met the inclusion criteria [136,137]. Sixteen of these studies were in vivo studies which described the anti-cancer effects of cannabinoids on glioma tumours [31]. Meanwhile, many in vitro and preclinical studies in animal models have successfully shown anti-cancer effects of cannabinoids based on reduction of tumour growth via the inhibition of tumour cell proliferation and angiogenesis, the tumour microenvironment, induction of tumour cell death, and inhibition of invasion through the genetic or pharmacological modulation of cannabinoid and other receptors [129,138,139,140,141,142]. A study assessing the need for the addition of serum to in vitro testing conditions of cannabinoids reaffirmed the importance of mimicking the tumour microenvironment in vitro and warned about the high degree with which cannabinoids bind to plastic in vitro [143]. This is because the tumour microenvironment is a complex and dynamic environment that can influence the efficacy of cannabinoids. By mimicking the tumour microenvironment in vitro, researchers can develop more accurate and predictive models of cannabinoid activity [144]. This can help to prevent clinical failure associated with differences between in vitro models and human subjects. A study investigated the in vitro and in vivo efficacy of cannabidiol (CBD) in neuroblastoma, a nervous system tumour in children [145]. Two cannabinoids, tetrahydrocannabinol (THC) and CBD, were experimentally tested to determine the effects of the compounds on invasiveness, programmed cell death, viability, and cell cycle distribution in human neuroblastoma cells in vitro. The cannabinoids were also evaluated for their ability to reduce the growth of tumour xenografts in vivo in mice. The results showed that both THC and CBD had antitumourigenic activity in vitro. However, CBD was more active than THC in reducing the invasiveness, apoptosis, viability, and cell cycle distribution of neuroblastoma cells. In vivo, CBD also showed greater efficacy than THC in reducing the growth of tumour xenografts in mice. Further studies are needed to confirm these findings and to evaluate the safety and efficacy of CBD in clinical trials. 

**Figure 5:**
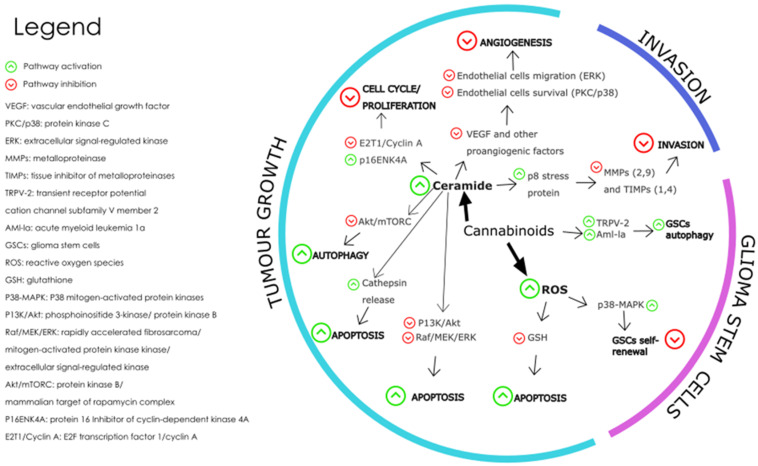
The main molecular mechanisms affected by cannabinoids during the modulation of GBM are depicted where the green arrows represent pathway activation and the red arrows represent pathway inhibition [146]. Redrawn with permission from Dumitru, C.A., Cannabinoids in Glioblastoma Therapy: New Applications for Old Drugs; published by Front Mol Neurosci, 2018. Creative Commons CC BY 4.0. http://creativecommons.org/licenses/by/4.0/ (accessed on 26 June 2023).

The levels of endocannabinoids and expression of their receptors present in glioblastoma microenvironment are dysregulated in the disease state and this dysregulation is thought to contribute to the growth and progression of GBM tumours [27]. Figure 5 shows the pathways triggered by cannabinoid receptor interaction which affect the hallmarks of cancer associated with glioblastoma tumours. 

The mechanisms involved in the effect of cannabinoids on GBM tumour growth include cell death-inducing mechanisms, anti-angiogenic mechanisms, and anti-proliferation mechanisms. Cannabinoid-induced cell death is prompted by the activation of intrinsic apoptosis pathway by cannabinoid-receptor interaction which results in increased intracellular ceramide, thereby inhibiting pathways PI3K/Akt and Raf1/MEK/ERK [124]. The PI3K/Akt/mTOR pathway is a key signalling pathway that regulates cell proliferation, survival, and metabolism. Activation of CB1 and CB2 receptors by cannabinoids has been shown to inhibit the PI3K/Akt/mTOR pathway in glioblastoma cells, leading to a decrease in cell proliferation and an increase in apoptosis and autophagy [147]. This effect is thought to be mediated by the inhibition of Akt phosphorylation and activation, as well as the downregulation of downstream targets such as mTOR, p70S6K, and 4EBP1 [148]. 

Cannabinoid induced apoptosis is also triggered by oxidative stress as seen when glioma cells treated with CBD caused an increase in reactive oxygen species (ROS) formation [149,150]. The MAPK/ERK pathway is another important signalling pathway that regulates cell proliferation, differentiation, and survival. Activation of CB1 and CB2 receptors by cannabinoid ligands have been shown to modulate the MAPK/ERK pathway in glioblastoma cells, leading to a decrease in angiogenesis and an increase in apoptosis [151,152]. This effect is thought to be mediated by the inhibition of ERK phosphorylation and activation, as well as the downregulation of downstream targets such as c-fos and c-jun. 

In a recent study, a standard mix of *cannabis* extracted active fractions F4 and F5 was found to induce apoptosis and expression of endoplasmic reticulum (ER)-stress associated-genes in glioblastoma cells [153]. The fractions F4 and F5 also inhibited cell migration and invasion, altered cell cytoskeletons, and inhibited colony formation in 2 and 3-dimensional models. The study suggests that combinations of cannabis compounds exert cytotoxic, anti-proliferative, and anti-migratory effects on glioblastoma cells. The JNK pathway is a stress-activated signalling pathway that regulates cell survival and apoptosis [154]. Activation of CB1 and CB2 receptors by cannabinoids has been shown to activate the JNK pathway in glioblastoma cells, leading to an increase in apoptosis [155]. This effect is thought to be mediated by the activation of JNK phosphorylation and the upregulation of downstream targets such as c-jun. Further research is needed to fully understand the molecular mechanisms of cannabinoid action in glioblastoma, as well as the potential for developing cannabinoid-based therapies for this deadly disease [156].

The physicochemical properties of most traditional cannabinoids, which include high lipophilicity, poor water solubility, and chemical instability, present significant formulation challenges for the development of effective therapies for brain tumours. However, advances in pharmaceutical science and technology are helping to overcome these challenges, and to harness the potential of cannabinoids for the treatment of brain tumours. The lipophilic nature of cannabinoids may be beneficial for cannabinoid delivery to the brain but tend to lend to the formation of colloidal aggregates which artifacts in early drug discovery and proves difficult to achieve suitable solubility and stability in aqueous solutions. However, they possess an attractive composition as nanoparticle formulations for targeted drug delivery. A combination of ligand proteins and polymers may be used to stabilise the colloidal aggregates, reduce colloid size, and improve longevity in blood circulation [157]. Besides the high hydrophobicity associated with most cannabinoids, including THC, the ability to elicit CB1-R mediated psychoactivity is one of the most noted drawbacks of cannabinoid therapeutic use [158,159]. All sources of evidence investigated in a recent study, including randomised controlled trials, observational studies, and Mendelian Randomisation studies have consistently indicated the use of cannabis is associated with an increased risk of psychosis and a potentially increased risk of psychiatric symptoms such as mania [160,161]. A systematic review of the safety of cannabinoids for medical use was conducted [162]. The review found that there is insufficient data on the safety of cannabinoids, but most studies reported no adverse events (AEs) with acute administration and mild to moderate AEs with chronic administration. The most common AEs reported were drowsiness, fatigue, and dry mouth [163]. An association between cognitive impairment and cannabis has been shown in observational studies and randomized controlled trials, which has also been associated with motor vehicle accidents [164]. While CBD has demonstrated promising efficacy in various clinical trials, it is essential to recognize its intrinsic pharmacological effects, potential adverse drug events, and the possibility of pharmacokinetic and pharmacodynamic drug-drug interactions [165]. Given the increasing prevalence of CBD use among patients with complex medical conditions and treatment regimens, as well as its widespread availability as a consumer product, a comprehensive understanding of CBD’s safety profile is paramount [166]. Further research is needed to better understand the safety of cannabinoids for medical use. 

## 6. Current Standard Treatment and Associated Challenges

The first-line therapy for primary glioblastoma is surgical intervention, preceded by chemo-radiation and chemotherapy. This treatment protocol leaves the disease as incurable while only extending survival time to 15 months after diagnosis [34,35]. The standard treatment in good performance patients follows the Stupp protocol for glioblastoma, whereby maximal safe tumour surgical resection is followed by post-operative ionising radiation (radiotherapy) alone or in concomitant use of temozolomide (TMZ) chemotherapy [40]. Temozolomide belongs to the imidazotetrazine class of compounds and functions as a prodrug that is converted to the active metabolite MTIC through spontaneous hydrolysis within the body. As an oral alkylating therapeutic, it is prescribed for the management of primary malignant glioblastoma multiforme in conjunction with radiotherapy, as well as for the treatment of malignant melanoma. The efficacy of temozolomide is attributed to its metabolites, which methylate guanine bases within the DNA. This methylation leads to DNA strand breaks, subsequently triggering cell death through apoptosis. The use of bevacizumab is considered in patients with neurological dysfunction secondary to tumour oedema [167,168]. Bevacizumab, a humanized monoclonal IgG1 antibody, targets and blocks VEGF-A, a protein that plays a crucial role in tumour growth and angiogenesis [169]. By selectively binding circulating VEGF-A, bevacizumab prevents it from interacting with its cell surface receptors, effectively halting the formation of new blood vessels that supply oxygen and nutrients to tumours [170]. This disruption of the tumour’s blood supply leads to reduced interstitial pressure within the tumour tissue, increased vascular permeability, and enhanced delivery of chemotherapeutic drugs. Additionally, bevacizumab promotes apoptosis further hindering tumour growth. In addition to these standard treatments, there are also several new and emerging treatments for glioblastoma that are currently being studied [171]. These include immunotherapy, which involves using the body’s own immune system to fight the cancer, and targeted therapy, which uses drugs that specifically target the cancer cells while sparing normal cells [172,173]. The presence of the blood brain barrier (BBB) and absence of lymphatic vessels in the brain, was the reason the immune system was previously neglected in the treatment of brain tumours. While surgery is often the first treatment option for GBMs, glioblastoma cells exhibit a diffuse invasion pattern causing tumour cells to infiltrate healthy brain tissue beyond the tumour margin, therefore it is often difficult to remove the entire tumour. After surgery, radiation therapy is often used to kill any remaining tumour cells, covering a margin of 2 cm beyond the visible tumour margin, although microscopic tumour invasion has often spread beyond this point. Tumour cells in infiltrating glioblastoma are enriched with glioblastoma stem cells (GSCs), a subset of tumour cells that can propagate and differentiate into various cell types, contributing to the extensive heterogeneity of GBM cell phenotypes. GSCs are highly resistant to chemotherapy, and their presence within tumour cells can drive tumour recurrence and the development of chemoresistance [36]. The high resistance to common chemotherapy and radiation is also attributed to inter- and intratumour heterogeneity where different genetic and molecular features cause different responses to therapy, the influence of the tumour microenvironment, and a varying array of mutations found in GBM that interfere with signalling pathways associated with DNA repair, cell proliferation, and survival [37,38,39]. The robust DNA repair and self-renewing capabilities of glioblastoma cells and glioma-initiating cells confer resistance to all current treatment modalities. Therefore, durable management of GBM will require the development of innovative therapeutic strategies [174,175]. The slow translation of preclinical acquisitions in the GBM field has prompted a plea from the scientific community to evaluate more appropriate treatment schedules and trial designs in the clinical setting. Methods such as introducing the therapeutic impact factor (TIF) and therapeutic (t) index bibliometric parameters to preclinical research are hypothesised to gear toward more therapy-focused activities [176].

The first reported pilot phase I clinical trial in humans, testing the antitumoural effect of cannabinoids in patients with recurrent glioblastoma multiforme, was undertaken by Guzman, 2006 [158]. Nine GBM patients were intratumourally administered THC in the pilot trial. The patients had previously experienced failed standard therapy, including surgery and radiotherapy while showing clear evidence of tumour progression. The safety and dosage of intracranial administration of THC were assessed, along with an evaluation of THC’s effect on the length of patient survival and other tumour-cell parameters. The cannabinoid delivery was safely achieved without overt psychoactive effects, and the median survival rate of the patients was 24 weeks (95% confidence interval: 15–33 weeks) since the start of cannabinoid administration. The study supported that THC did not facilitate tumour growth nor decreased patient survival in GBM patients expressing cannabinoid receptors. 

Torres, 2011 showed that the combined administration of THC and TMZ exerted a robust antitumoural action in glioma xenografts [177]. This effect was also present in tumours that were resistant to TMZ treatment. This work demonstrated that the coadministration of THC and TMZ strongly enhanced autophagy in vitro human glioma cell lines and in vivo tumour xenografts of mice. The combination of THC and TMZ significantly reduced tumour growth in the animal model compared to the individual agents. The study also tested a combination of TMZ with submaximal doses of THC and CBD, where significant antitumoural action was produced in both TMZ-sensitive and TMZ-resistant tumours. Another recent preclinical study investigating the optimisation of cannabinoids in combination with TMZ against glioma showed that at a 1:1 ratio of THC and CBD in combination with TMZ. A profound antitumoural effect was seen in both subcutaneous and intracranial glioma cell-derived tumour xenografts [178]. The authors argue that the potential utilisation of cannabinoids as anti-cancer agents depends on whether effective doses of the cannabinoids can be achieved in human GBM patients. A dose conversion estimates that while doses of THC and CBD between 5 and 10 mg/kg per day are adequate for anti-cancer activity in mice, in humans, this dose will be equivalent to an administration of 25 to 50 mg of THC and CBD per day. A phase Ib randomized, placebo-controlled clinical trial using cannabinoid oromucosal spray, nabiximols with temozolomide in patients with recurrent GBM showed that with personalized dosing, nabiximols presented acceptable safety and tolerability without any drug–drug interactions identified [179]. Nabiximols (Sativex), a cannabinoid-based medication composed of equal parts THC and CBD, is an approved treatment for spasticity, a symptom characterized by muscle stiffness and spasms in adults with multiple sclerosis [180]. Available as an oromucosal spray, Nabiximols has garnered regulatory approval in various countries [181]. The clinical trial (Part 1—NCT01812603; Part 2—NCT01812616) observed survival differences that require further exploration in a more adequately powered randomized controlled trial [177,178].

The brain tumour microenvironment presents unique challenges for treatment due to several distinctive features, including the blood–brain barrier (BBB), the presence of interconnected and myelinated axon tracts, and a specific ECM composition [182]. Even when compromised during tumour progression, the BBB remains largely impermeable to most chemotherapeutic agents, especially in the actively invading tumour regions where the BBB is intact [183]. In addition, vascular basement membrane haptotactic cues and vascular-derived chemotactic cues can further drive tumour cell invasion and therapeutic resistance in the perivascular space [184]. Interconnected axon tracts also provide haptotactic cues that contribute to cellular invasion and present a significant challenge to surgical resection. Furthermore, the brain ECM is softer (300–3000 kPa), contains less collagen fibres, and is enriched in hyaluronic acid (HA), tenascins, and chondroitin sulfates, which can impact treatment efficacy [185]. Three main barrier systems protect the brain from circulating pathogens and toxic substances and support neuronal function by maintaining ionic homeostasis and nutrient supply: the blood–brain barrier, the meningeal barrier, and the blood cerebrospinal fluid barrier [186]. The formulation of small hydrophobic drug molecules is a challenge in developing new treatment and poses a barrier between drug development and clinical use. More than 98% of small molecule drugs are unable to cross the barriers to enter the CNS [187]. The Lipinski rule of five, a widely used tool for assessing druglikeness, evaluates the likelihood of a compound’s oral bioavailability and BBB penetration [188]. Formulated by Christopher A. Lipinski in 1997, the rule is based on the observation that most orally active drugs possess relatively small molecular sizes and moderate lipophilicity [189]. The rule consists of four parameters: no more than five hydrogen bond donors, no more than ten hydrogen bond acceptors, a molecular mass below 500 daltons, and an octanol-water partition coefficient (clogP) not exceeding 5. While not a definitive predictor of druglikeness, the Lipinski rule of five serves as a valuable tool for identifying drug candidates with potentially poor oral absorption and the likelihood of a small molecule passing through the BBB [190]. 

## 7. Novel Pharmaceutical Anti-Cancer Strategies to Overcome GBM Treatment Challenges

The BBB and brain–tumour barrier (BTB) have been identified as causes of poor treatment response due to the limitations associated with penetration of antineoplastic drugs into the brain [191]. Direct drug injections via the intranasal or intrathecal routes, chemical modification of drugs or BBB constituents, inhibition of efflux pumps, physical disruption of the BBB by radiofrequency electromagnetic radiation, laser-induced thermal therapy, focused ultrasounds combined with microbubbles, and convection enhanced delivery are all methods used to circumvent the BBB’s limitations [192]. The choroid plexus, which is the interface between the blood and cerebrospinal fluid barriers, is an epithelial boundary that can be exploited for drug delivery to the brain by way of cerebrospinal fluid [193,194]. Immunohistochemistry revealed that CB1-R protein as well as FAAH are expressed in the rat choroid plexus epithelia [195]. The highly aggressive glioblastoma tumour has been linked to *c-myc* which is a proto-oncogene that is often elevated in its malignancy. Intranasal delivery of genes to the brain is another promising approach to interfere with *c-myc* expression, but effective delivery to glioma cells and avoidance of premature release during transport is essential for therapeutic efficacy. To address these challenges, the authors of a recent study constructed a stable lipoplex based on pre-compressed *c-myc*-targeting *siRNA* encapsulated by liposomes modified with a selected peptide [196]. The lipoplex was preferentially internalised by glioma cells via active macropinocytosis, avoiding lysosomal entrapment and releasing *siRNA* within 4 h to induce substantial downregulation of *c-myc* mRNA and protein expression. The lipoplex also exhibited enhanced permeability in tumour spheroids and nasal mucosa, delivering more siRNA to orthotopic glioma after intranasal administration and prolonging the survival time of glioma-bearing mice by inducing apoptosis.

**Figure 6:**
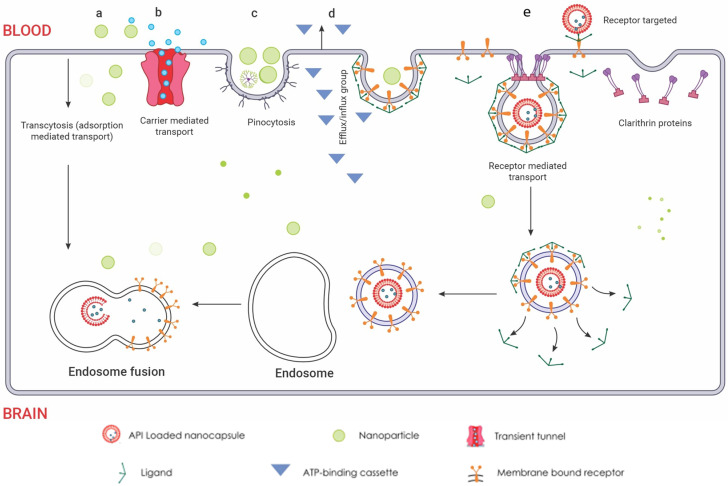
Routes of nanoparticle delivery across the BBB: (**a**) Cellular adsorption-mediated transport. Harnessing the inherent physicochemical properties of naturally sourced materials, the fabrication of surface-charge-tailored nanoparticles 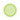 allows for targeted cellular internalization via adsorption-driven processes. (**b**) Carrier-mediated transport. This mechanism leverages the inherent selectivity of endothelial transporters, akin to molecular gatekeepers, for targeted nanoparticle internalisation. Functioning like protein shuttles, these transporters recognize specific ligands displayed on nanoparticle surfaces 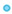, facilitating their entry across the BBB endothelium. This selective binding triggers conformational changes in the transporter, creating a transient tunnel 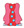 for the nanoparticle to bypass the tight junctions and enter the brain parenchyma. This enables precise delivery of encapsulated drugs directly to neuronal targets, bypassing the restrictive diffusion limitations and efflux pumps that pose significant challenges for conventional drug delivery. (**c**) Pinocytosis transport. Specific surface features on the nanoparticles are recognized by receptors, triggering membrane invagination and vesicle formation. This targeted transport offers advantages like enhanced uptake and potential for organelle-specific delivery, while presenting challenges like non-specificity and saturation. (**d**) Efflux pump mechanism. These pumps, embedded in the endothelial membrane, recognize specific chemical features on the nanoparticle surface, triggering conformational changes that propel the nanoparticle out of the cell. The membrane-bound ATP-binding cassette 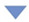 transporters efflux mechanism utilizes conformational changes within the transporter protein to generate a directional transport pathway, safeguarding the brain from potentially harmful substances. This efflux mechanism presents a formidable challenge for nanoparticle delivery, hindering their ability to reach their target sites within the brain. However, understanding the selectivity and mechanisms of efflux pumps can guide the design of nanoparticles with optimized surface properties, enabling them to evade detection and bypass this cellular blockade. (**e**) Receptor-mediated endocytosis. Nanoparticles functionalized with transferrin or LDL ligands 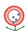 engage in specific, high-affinity interactions with cognate receptors on the endothelial cell surface. This ligand-receptor binding triggers clathrin-mediated pit formation 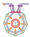, resulting in the encapsulation of the nanoparticle within a membranous vesicle. This vesicle then undergoes scission and vesicular transport, delivering the nanoparticle directly into the brain parenchyma. Within the brain parenchyma, these nanoparticle-laden vesicles fuse with early endosomes 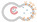, initiating the intracellular sorting process. Early endosomes contain specific receptors and sorting molecules that recognize the nanoparticle’s surface ligands and direct its trafficking towards distinct intracellular compartments. The acidic lumen of the early endosome triggers ligand dissociation from the nanoparticle, allowing for potential ligand recycling and further processing of the nanoparticle. Redrawn with permission from Ndemazie, N.B., Multi-Disciplinary Approach for Drug and Gene Delivery Systems to the Brain. AAPS; published by PharmSciTech, 2022. “Methods of transporting materials across the BBB” by Ndemazie, et al. is licensed under CC BY 4.0 [206]. http://creativecommons.org/licenses/by/4.0/ (accessed on 22 July 2023).

Nanoparticles (NPs) or drug-delivery nanosystems are being extensively studied as a promising strategy for direct drug delivery to the CNS. NPs have the potential to maintain therapeutic drug levels, increase drug stability and solubility, and effectively cross the blood–brain barrier for treating brain diseases. In preclinical settings, researchers have evaluated a diverse array of NP systems for GBM drug delivery, encompassing lipid-based NPs, polymeric NPs, dendrimers, micelles, and inorganic NPs like gold, silica, and quantum dots [197,198,199,200]. These systems differ in crucial physicochemical properties such as size, composition, shape, and surface attributes, which dictate their ability to traverse the BBB, navigate the brain’s intricate microenvironment, and specifically target and enter diseased cells. An excellent review on the preclinical studies of nanotherapeutics for GBM suggests promising benefits. Translating this efficacy to humans remains a challenge [201]. Current animal models often poorly mimic human GBM pathology and BBB transport, limiting their predictive power [202]. However, new models like brain organoids and lab-on-chip BBB models, using human-derived cells, offer more accurate reflections of the complex tumour microenvironment and BBB [203,204]. These models hold immense potential for personalized medicine, as demonstrated by the successful use of patient derived GBM organoids for *ex vivo* drug screening. In essence, the future of nanotherapeutic development for GBM lies in advanced, human-relevant models that bridge the gap between preclinical and clinical settings. The uptake of NPs into the brain is believed to occur through adsorptive transcytosis and receptor-mediated endocytosis, depending on particle characteristics, shown in Figure 6. Current research focuses on incorporating multiple functionalities and moieties in drug-delivery nanosystems to overcome barriers and achieve site-specific accumulation of nanotherapeutics at diseased sites, making site-specific drug delivery a significant advancement in modern nanotherapeutic design. Organic-based biodegradable polymers, often derived from natural or semisynthetic sources, are increasingly being used to prepare formulations for nanotherapeutics [205]. These polymers are capable of degrading in vivo into safe, biocompatible products that are eliminated from the body through standard metabolic pathways. Many such materials have been used as excipients or matrices for controlled drug delivery. Encapsulation technology is used to design controlled release nanocarriers based on these matrices.

The physical, chemical, and biopharmaceutical characteristics of CBD have been investigated in several studies [207]. The physicochemical characteristics of cannabinoids which contribute towards challenges associated with cannabinoid delivery include low aqueous solubility (2–10 µg/mL) and susceptibility to auto-oxidation and degradation caused by changes in light or temperature [208,209]. The U.S. Food and Drug Administration (FDA) has approved cannabis-related products (e.g., Epidiolex, Syndros, Cesamet and Marinol), while research in this area is ongoing [210]. Since the approved products are in oral dosage form, which can limit their therapeutic efficacy, nanotechnology is being investigated as a potential method to improve the bioavailability and therapeutic efficacy of cannabis products [211]. Nanocarrier technology has been used to enhance the efficacy, stability, release and biopharmaceutical interaction of natural and synthetic cannabinoids, and formulation, while preclinical and clinicals studies have also been performed with cannabinoid-based nano colloidal carriers to investigate biocompatibility, bioavailability, and solubility [212,213,214]. Cannabinoid receptors’ structural and functional properties have been investigated to facilitate specific drug and probe drug delivery designs that enable precise modulation of the ECS via cannabinoid receptor–ligand interactions and the activation and signalling of cannabinoid receptors with functional and subtype receptor selectivity [215]. To gain a deeper understanding of CB2-R-ligand complex activation pathways, a study on pharmacological and imaging tools including covalent selective CB2-R ligands, photochromic, fluorescent, and positron emission tomography ligands was done to assist in designing suitable CB2-R-targeted drug delivery systems [216]. CBD is a small, water-insoluble molecule with a reported water solubility of 12.6 mg/L, and it is lipophilic with a log *p* value of 6.3 [217]. CBD is unstable in gastric pH, susceptible to first-pass metabolism, and has low susceptibility to renal excretion [218]. As a result, CBD is classified as a Class II drug according to the Biopharmaceutical Classification System and the Biopharmaceutical Drug Classification System, due to its low water solubility and high permeability. CBD is also classified as a Class II compound by the Biopharmaceutics Drug Disposition Classification System due to its low water solubility and metabolic elimination. The low and unpredictable oral bioavailability of CBD further complicates formulation development, likely due to these properties [219]. Advanced drug delivery systems have been investigated to improve the CBD dissolution profile, produce a site-specific release, protect against metabolisation, and increase its bioavailability [220]. 

Targeted drug delivery using the ECS holds immense promise for developing innovative and intelligent therapies. By directly delivering cannabinoids to the site of action, targeted drug delivery systems can enhance pharmaceutical specificity, minimise side effects, and overcome formulation challenges associated with cannabinoids. Recent advancements in nanotechnology have paved the way for a diverse array of targeted drug delivery strategies for the ECS [221]. Understanding the intricate signalling pathways, distribution, receptor structure, and enzymatic degradation of the ECS is crucial for designing optimised nanocarriers [222]. Homology modelling can provide deeper insights into the therapeutic potential of the ECS toolkit. Nanotechnology and surface modification techniques offer promising avenues for specific targeting of ECS-related therapeutics [223]. Further research is warranted to translate these technologies into clinical practice and revolutionise the treatment of various diseases.

Lipid nanocapsules (LNCs) decorated and loaded with CBD for glioma therapy were prepared and their critical parameters evaluated in vitro in a recent study [224]. CBD was encapsulated into the oily core of LNCs to test their extended-release efficacy against U373MG human glioblastoma cells. The antitumour effect of CBD-loaded LNCs was highly dependent on the size of LNCs, with smaller LNCs (20-nm) reducing the IC50 value by 3.0-fold compared to their 50-nm counterparts. CBD-functionalisation of LNCs significantly enhanced glioma targeting by 3.4-fold compared to undecorated LNCs of the same size, indicating the potential of CBD to target cannabinoid receptors overexpressed in glioma cells. Furthermore, the combination of CBD-loading with CBD-functionalisation further reduced the IC50 values. These findings highlight the potential of CBD-loaded LNCs as a promising approach for glioma therapy and warrant further in vivo evaluation of these strategies for CBD incorporation into LNCs.

In the study of Li, 2022, researchers synthesised betulinic acid nanoparticles (BA NPs) and evaluated their antitumour effects in vitro and in vivo. BA NPs were successfully prepared and were found to be effective at suppressing glioma cell proliferation, inducing apoptosis, and arresting the cell cycle in the G0/G1 phase while also downregulating the Akt/NFκB-p65 signalling pathway [225]. In addition, BA NPs were able to cross the BBB in mice with GBM tumours, and the NPs were able to prolong the survival time of the mice by an average of 40%. A study investigating the use of biobased poly(thioether-ester) nanoparticles (PTEe NPs) to encapsulate full-spectrum cannabis extract (CN) and improve its bioavailability, showed via in vitro studies that CN-PTEe NPs were able to reduce the viability of cancer cells (B16F10, T98, and U87) to a similar extent as free CN [226]. Two different methods were used to synthesise the PTEe NPs: *in-situ* thiol-ene miniemulsion polymerisation (Me-PTEe) and the thiolene miniemulsification/solvent evaporation method using PTEe synthesised previously by thiolene bulk polymerisation (Se-PTEe). The results showed that both methods were effective in producing CN-PTEe NPs with high encapsulation efficiency. The NPs had an average diameter of between 91 and 229 nm and were non-toxic to non-cancerous cells.

## 8. Conclusions

Despite the use of current therapeutics and recent advances in molecular pathology, GBM remains a fatal disease with an incredibly poor prognosis that requires an urgent need to target the molecular mechanisms owing to its progression. Studies indicate that patients survive approximately 15 months after diagnosis and that the clinical and biological factors which affect survival include histologic findings, age at presentation, molecular genetic factors, the size and location of tumour, and therapeutic approaches undertaken. Glioblastoma may present as a difficult disease to treat due to its diversity, cellular and tissue biology, genetics, pathophysiology, and therapeutic responses. Prospective treatments are based on powerful experimental and computational tools that provide an avalanche of ‘big data’ that encompasses a myriad of indicators of the disease [94]. This interdisciplinary approach assists in distilling the complexity of the hallmarks of cancer into a logical science to gain new perspectives and a fuller understanding of the mechanisms of cancer development and malignant progression with the main goal of designing more sophisticated glioblastoma treatment. Future treatment must focus on designing molecularly targeted drug delivery systems based on deep knowledge established from pathophysiological mechanisms [95]. Looking ahead, suitable treatment must not only overcome drug and radio resistance, but it must also aim to avoid side effects and relapses. The topic of cannabinoids in the treatment of glioblastoma is still in its infancy. While there is some evidence to suggest that cannabinoids may be effective adjuvants to the current treatment protocol, more research is needed to establish them as anti-cancer agents for GBM. The gold standard for treatment of GBM remains surgery, followed by chemo- and radiation therapy. However, these treatments can be ineffective due to chemoresistance and can also have significant side effects. Cannabinoids may offer a more effective and tolerable treatment option for GBM patients. The significant potential drawbacks of most cannabinoid utilisation, such as psychoactive effects associated with CB1-R activation and the potential for abuse, may play a role in the reluctance of human clinical trials. However, the recent advancement of innovative drug delivery systems may mitigate the risk of these drawbacks and thereby increase the human clinical research in this field. Overall, the research on cannabinoids in the treatment of GBM is promising. However, more research is needed to determine their safety and efficacy. Guided by these findings, we envision future research delving into the precise signalling pathways within the endocannabinoid system that mediate glioblastoma’s response to hormonal, immunological, and mechanical cues. Additionally, investigating the potential for synergistic combination therapies that integrate endocannabinoid-based interventions with existing treatment modalities could offer promising avenues for improved patient outcomes. While this work sheds light on the intriguing potential of the endocannabinoid system in glioblastoma management, significant challenges remain in translating these findings to the clinic. Future research must address issues of drug delivery, potential off-target effects, and interpatient variability in response to cannabinoid-based therapies to fully realize the clinical potential of this novel approach.

## Figures and Tables

**Table 1:** Nomenclature-derived cell of origin of associated brain tumours.

Cell Type		Associated Tumours
Astrocyte	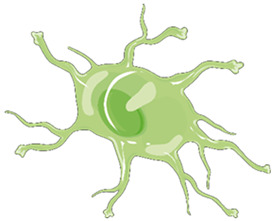	Astrocytoma Pilocytic astrocytoma (grade I) Diffuse astrocytoma with IDH mutations (grade II) Anaplastic astrocytoma (grade III)
Oligodendrocyte	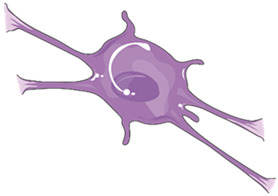	Oligodendrogliomas
Ependymal cells	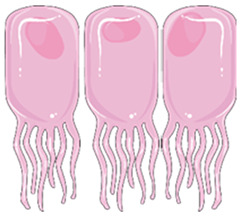	Ependymomas
Microglia	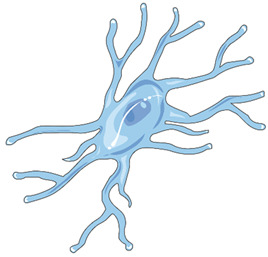	Glioblastomas IDH-Wildtype (grade IV astrocytoma) [46]
Neural stem cells	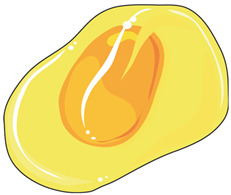	

**Table 2:** The key genotypic markers used for WHO categorisation are explained.

Molecular genotypic key markers explained	
IDH	IDH enzymes are involved in the citric acid cycle. Mutations in the genes coding for IDH1 and IDH2 are often found in gliomas and result in a neomorphic enzyme activity that produces 2-hydroxyglutarate, an oncometabolite [50]. This accumulation can lead to a hypermethylation phenotype and alterations in cell differentiation, contributing to oncogenesis.
p53 Mutations	The TP53 gene, which encodes the p53 protein, role in regulating the cell cycle and maintaining genomic stability [51]. Mutations in p53 are one of the most common genetic alterations in human cancers, leading to loss of function and allowing cells to proliferate unchecked.
ATRX	ATRX mutations are often present in gliomas and are associated with alternative lengthening of telomeres, a telomerase-independent mechanism for telomere maintenance [52]. These mutations can lead to genomic instability and have been linked with a specific subtype of gliomas that have a particular molecular signature and prognosis [53].
1p/19q Chromosomal Deletion	This co-deletion is a hallmark of oligodendrogliomas and is associated with a better response to chemotherapy and radiotherapy, as well as a more favourable prognosis [54]. The loss of these chromosome arms is believed to lead to the loss of tumour suppressor genes, although the precise mechanism by which this improves treatment response is not fully understood.
H3K27M	H3K27M mutations are characteristic of diffuse intrinsic pontine gliomas and some midline gliomas [55]. This mutation results in a gain-of-function that affects the methylation status of histone H3, thereby altering gene expression. These mutations are associated with a more aggressive disease course and have been recognized as a distinct entity in the WHO classification of tumours of the central nervous system.

**Table 4:** Cannabinoids: A Classification Based on Structural Features and Pharmacological Effects.

**Classical Cannabinoids**
Classical cannabinoids are the most well-known group of cannabinoids, and they are found in the cannabis plant. They have a highly lipophilic structure and poor water-solubility due to their characteristic tricyclic terpenophenolic structure [109]. This lipophilicity facilitates easy passage through the lipid bilayers of cell membranes, influencing their absorption and distribution. Classical cannabinoids are extensively metabolised in the liver, primarily by cytochrome P450 enzymes, leading to a variety of metabolites, some of which are active and contribute to its pharmacological effects [110,111]. The high lipophilicity and poor water solubility of classical cannabinoids pose challenges in formulating them for aqueous-based delivery systems [112]. Techniques like nanoemulsions, liposomes, or microencapsulation may be employed to enhance solubility and bioavailability. Examples: THC, CBD, CBN
**Non-Classical Cannabinoids**
Non-classical cannabinoids, often synthetic cannabinoids that are not found in the cannabis plant, can be designed to have specific physicochemical properties [113]. They may be more potent and selective for cannabinoid receptors than classical cannabinoids [114]. They may be designed to have increased metabolic stability, thereby prolonging their duration of action [115]. However, their synthetic nature might lead to unpredictable metabolism and potential toxic metabolites. Formulation strategies would depend on the specific properties of the compound. Solubility enhancement and targeted delivery systems could be key considerations. Examples: CP 47497, CP 55940
**Aminoalkylindoles**
Aminoalkylindoles have a simpler, more stable structure compared to classical cannabinoids. The aminoalkylindole chemical class can be subdivided into four groups: naphthoylindoles, phenylacetylindoles, benzoylindoles, and naphthylmethylindoles [116]. This influences their interaction with cannabinoid receptors, making them more selective for cannabinoid receptors [117]. These compounds generally have high lipophilicity and may show significant brain penetration due to their ability to cross the blood–brain barrier efficiently. Similar to classical cannabinoids, addressing solubility and stability issues is critical [118]. There’s also a need to consider the potential for rapid onset of action due to efficient CNS penetration. Examples: WIN-55212-2, JWH-018
**Eicosanoids**
Endocannabinoids including anandamide and 2-AG are derived from fatty acids, making them lipophilic structures [119,120]. This allows easier cellular uptake and interaction with cannabinoid receptors [121]. Endocannabinoids are rapidly metabolised in the body, which can limit their therapeutic use unless modifications or delivery systems are employed to stabilise them [122]. Enhancing stability and prolonging the duration of action are primary goals. Techniques might include the use of enzyme inhibitors to prevent rapid degradation or using advanced delivery systems to target specific tissues. Examples: Anandamide, 2-AG

## Data Availability

No new data were created or analyzed in this study. Data sharing is not applicable to this article.

## Abbreviations

GBM	Glioblastoma
CNS	Central nervous system
WHO	World health organisation
TME	Tumour microenvironment
ECS	Endocannabinoid system
DNA	Deoxyribonucleic acid
RNA	Ribonucleic acid
BBB	Blood brain barrier
BTB	Brain tumour barrier
ECM	Extracellular matrix
MDSC	Myeloid-derived suppressor cells
TNFα	Tumour necrosis factor alpha
IL-6	Interleukin 6
IL-17	Interleukin 17
LPS	Lipopolysaccharide
p53	Tumour protein 53
CB1-R	Cannabinoid receptor type 1
CB2-R	Cannabinoid receptor type 2
FAAH	fatty acid amide hydrolase
MAGL	Monoacylglycerol lipase
IDH	iso-citrate dehydrogenase
ATRX	ATP-de-136 pendent helicase
H3K27M	Lys-27-Met mutations in histone 3
cIMPACT NOW	Consortium to 164 Inform Molecular and Practical Approaches to CNS Tumour Taxonomy
VEGF	vascular endothelial growth factor
LPS	lipopolysaccharide
GIT	Gastrointestinal tract
MGMT	O6-methylguanine-DNA methyltransferase
GATA6	GATA binding protein 6
CASP8	caspase-8
PI3K	phosphoinositide 3-kinase
Akt	protein kinase
MAPK	mitogen-activated protein kinases
ERK	extracellular signal-regulated kinase
JNK	c-Jun N-terminal kinase
p70S6K	70-kDa ribosomal protein S6 kinase
4EBP1	Eukaryotic initiation factor 4E-binding protein 1
ROS	reactive oxygen species
FAK	focal adhesion kinase
ER	endoplasmic reticulum
TMZ	temozolomide
GSCs	glioblastoma stem cells
TIF	therapeutic impact factor
HA	hyaluronic acid
NPs	nanoparticles
FDA	Food and Drug Administration
LNCs	lipid nanocapsules
BA NPs	betulinic acid nanoparticles
PTEe NPs	poly(thioether-ester) nanoparticles
CN	cannabis extract
Me-PTEe	miniemulsion polymerisation poly(thioether-ester) nanoparticles
Se-PTEe	thiol-ene bulk polymerisation polymerisation poly(thioether-ester) nanoparticles
